# The glycoimmune checkpoint receptor Siglec-7 interacts with T-cell ligands and regulates T-cell activation

**DOI:** 10.1016/j.jbc.2023.105579

**Published:** 2023-12-21

**Authors:** Natalie Stewart, John Daly, Olivia Drummond-Guy, Vignesh Krishnamoorthy, Jessica C. Stark, Nicholas M. Riley, Karla C. Williams, Carolyn R. Bertozzi, Simon Wisnovsky

**Affiliations:** 1 Faculty of Pharmaceutical Sciences, University of British Columbia, Vancouver, British Columbia, Canada; 2 Department of Chemistry & Sarafan ChEM-H, Stanford University, Stanford, California, USA; 3 Department of Biological Engineering, Massachusetts Institute of Technology, Boston, Massachusetts, USA; 4 Department of Chemical Engineering, Massachusetts Institute of Technology, Boston, Massachusetts, USA; 5 Koch Institute for Integrative Cancer Research, Massachusetts Institute of Technology, Boston, Massachusetts, USA; 6 Department of Chemistry, University of Washington, Seattle, Washington, USA; 7 Howard Hughes Medical Institute, Stanford, California, USA

**Keywords:** glycobiology, immunology, lectins, sialic acid, T-cells, Siglecs

## Abstract

Siglec-7 (sialic acid–binding immunoglobulin-like lectin 7) is a glycan-binding immune receptor that is emerging as a significant target of interest for cancer immunotherapy. The physiological ligands that bind Siglec-7, however, remain incompletely defined. In this study, we characterized the expression of Siglec-7 ligands on peripheral immune cell subsets and assessed whether Siglec-7 functionally regulates interactions between immune cells. We found that disialyl core 1 O-glycans are the major immune ligands for Siglec-7 and that these ligands are particularly highly expressed on naïve T-cells. Densely glycosylated sialomucins are the primary carriers of these glycans, in particular a glycoform of the cell-surface marker CD43. Biosynthesis of Siglec-7-binding glycans is dynamically controlled on different immune cell subsets through a genetic circuit involving the glycosyltransferase GCNT1. Siglec-7 blockade was found to increase activation of both primary T-cells and antigen-presenting dendritic cells *in vitro*, indicating that Siglec-7 binds T-cell glycans to regulate intraimmune signaling. Finally, we present evidence that Siglec-7 directly activates signaling pathways in T-cells, suggesting a new biological function for this receptor. These studies conclusively demonstrate the existence of a novel Siglec-7-mediated signaling axis that physiologically regulates T-cell activity. Going forward, our findings have significant implications for the design and implementation of therapies targeting immunoregulatory Siglec receptors.

The Siglecs (sialic acid-binding immunoglobulin-like lectins) are a family of immune receptors characterized by their shared affinity for carbohydrate ligands (glycans) that contain the sialic acid monosaccharide (1, 2, 3). In humans, there are 15 Siglec family members that are expressed primarily by innate immune cells (1, 2, 3). Most members of the Siglec family are inhibitory receptors (1, 2, 3). In these cases, binding between a Siglec and its ligands triggers phosphorylation of intracellular immunoreceptor tyrosine-based inhibitory/switch (ITIM/ITSM) motifs (1, 2, 3) (Fig. 1*A*). Subsequent recruitment of the tyrosine phosphatase SHP-1 to the cell surface delivers an inhibitory signal that suppresses immune cell activity (1, 2, 3). In recent years, there has been significant interest in Siglecs as targets for cancer immunotherapy (4). A wide range of tumor cells overexpress sialic acid–containing glycans (5, 6), and Siglec receptors are frequently upregulated on tumor-associated immune cells isolated from several types of cancer patient–derived samples (7, 8, 9, 10). Blockade of Siglecs with targeted antibodies, enzymatic removal of cell-surface sialoglycans, and targeted suppression of sialic acid synthesis have all been shown to activate potent anticancer immune responses in recent years (7, 8, 9, 10).

**Figure 1:**
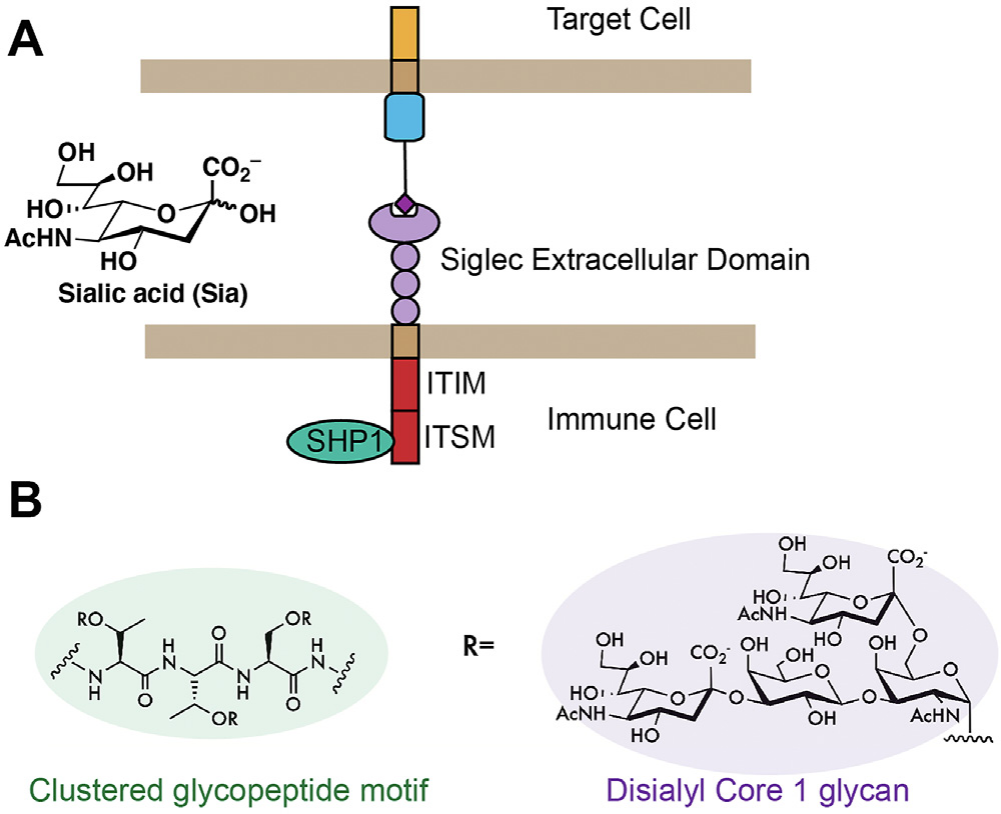
**Siglec-7 and its ligands.***A*, a Siglec immune receptor engages a glycan ligand. Signaling is then transduced through intracellular ITIM/ITSM domains. *B*, chemical structure of the putative Siglec-7-binding motif. Multiple disialyl core 1 O-glycans are arranged in clusters on amino acid residues directly adjacent to one another. This motif is found on specific densely glycosylated O-glycoproteins. Siglec, sialic acid–binding immunoglobulin-like lectin 7.

Despite this interest, the specific cell-surface ligands that bind each Siglec family member remain incompletely annotated. There are hundreds of distinct glycan structures that contain sialic acid. The relative affinities of different Siglecs for these glycans has been extensively studied *in vitro* using glycan microarray technology (1, 2, 3). Siglec-binding preferences have been found to vary significantly depending on the monosaccharide composition and linkage stereochemistry of the appended glycan (1, 2, 3). More recent studies have investigated interactions between Siglecs and their ligands in intact cells (11, 12, 13, 14, 15, 16, 17). These experiments have shown that some Siglecs can exhibit preferential binding to specific glycosylated proteins (11, 12, 13, 14, 15, 16, 17). The biochemical basis for this specificity can be hard to pin down. It is possible that some proteins may contain “Siglec-binding domains”: glycopeptide motifs where glycans are arranged on the protein backbone in a way that facilitates high-affinity binding (16, 18). Going forward, research to precisely characterize these motifs on different cell types may provide new insights into Siglec function (8).

In a recent study, we used a cell-based genomic screening approach to map genetic pathways involved in presentation of ligands for a particular Siglec (Siglec-7) on K-562 chronic myeloid leukemia cells. Downstream work revealed that Siglec-7 binds to an O-linked tetrasaccharide (the “disialyl core 1” antigen). Subsequent studies showed that ablating expression of this glycan stimulated immune killing of leukemia cells in both cell and animal models (18, 19, 20). In addition, we found that Siglec-7 binding to disialyl core 1 is influenced by the protein scaffold on which these glycans are presented. Siglec-7 exhibited particularly high binding to the N-terminus of a specific cell-surface marker called CD43. This glycopeptide region contains adjacent clusters of disialyl core 1 O-glycan tetrasaccharides that are closely spaced together on adjacent amino acids (Fig. 1*B*). Deletion of these motifs was found to abolish Siglec-7 binding (18). Another study has since confirmed that CD43 is the predominant ligand for Siglec-7 in K-562s (21). Independent work in glycoengineered human embryonic kidney 293 cells and patient-derived chronic lymphocytic leukemia cells similarly showed that Siglec-7 binds disialyl core 1 O-glycans on a variety of cell types (16, 22). The specific scaffolds on which these glycans are presented seems to vary in a cell–dependent manner. For example, a study in multiple myeloma uncovered other Siglec-7-binding proteins (*e.g.*, PSGL-1) with similar glycosylation and molecular features to CD43 (23). Taken together, these studies have collectively confirmed that Siglec-7 binds to mucin-type glycoprotein ligands that are densely glycosylated with disialyl core 1 O-glycans.

Building on these insights, we subsequently have become interested in defining the function(s) of the Siglec-7–disialyl core 1 interaction in immune regulation. Most prior work on Siglec-7 has focused on its role in suppressing the cytotoxic activity of innate immune cells toward cancer cell targets that express Siglec-7-binding glycans (2, 24). Characterization of Siglec-7 ligands has thus largely been confined to immortalized cancer cell lines. We hypothesized, however, that our findings may also have implications for understanding other aspects of the immunological function of Siglec-7. CD43, for example, is not a leukemia-restricted antigen. It is a physiological hematopoietic marker that is abundantly expressed on multiple peripheral immune cell types (25, 26, 27). Indeed, some studies have reported that murine immune cells express a glycoform of CD43 bearing disialyl core 1 tetrasaccharides (25, 26, 27). This is the same structure we and others have defined as a Siglec-7 ligand on cancer cell lines. In general, sialic acid-containing glycans are known to be expressed on peripheral immune cells (24, 28) and have been shown to be crucial for regulating numerous aspects of immune activation (29, 30, 31). No prior work, however, has specifically explored whether Siglec-7 binds to disialyl core 1 O-glycans/CD43 on healthy immune cells or what the immunological consequences of that interaction might be.

In this study, we broadly profile expression of Siglec-7 ligands on multiple peripheral immune cell subsets. We find that these ligands are specifically and abundantly expressed on primary T-cells. We then use a variety of assays to show that CD43 and other mucin-type glycoproteins bearing disialyl core 1 O-glycans are the predominant ligands for Siglec-7 on these cell types. We also show that Siglec-7 binds selectively to CD43^Di^^S^^iaCore1^ but not to other CD43 glycoforms expressed in the immune system. Relative levels of these O-glycans on different cell types are partially controlled by transcriptional activation/repression of the glycosyltransferase GCNT1. We demonstrate that interactions between Siglec-7 and T-cell ligands modulate T-cell activation and polarization in primary co-culture assays. Finally, we present evidence that in addition to acting as an inhibitory receptor on myeloid cells, engagement of T-cell ligands by Siglec-7 may also directly stimulate signaling pathways within T-cells. Taken together, these data delineate a novel role for Siglec-7 in regulating intra-immune signaling.

## Results

### Siglec-7 ligands are highly expressed on peripheral T-cells

**Figure 2:**
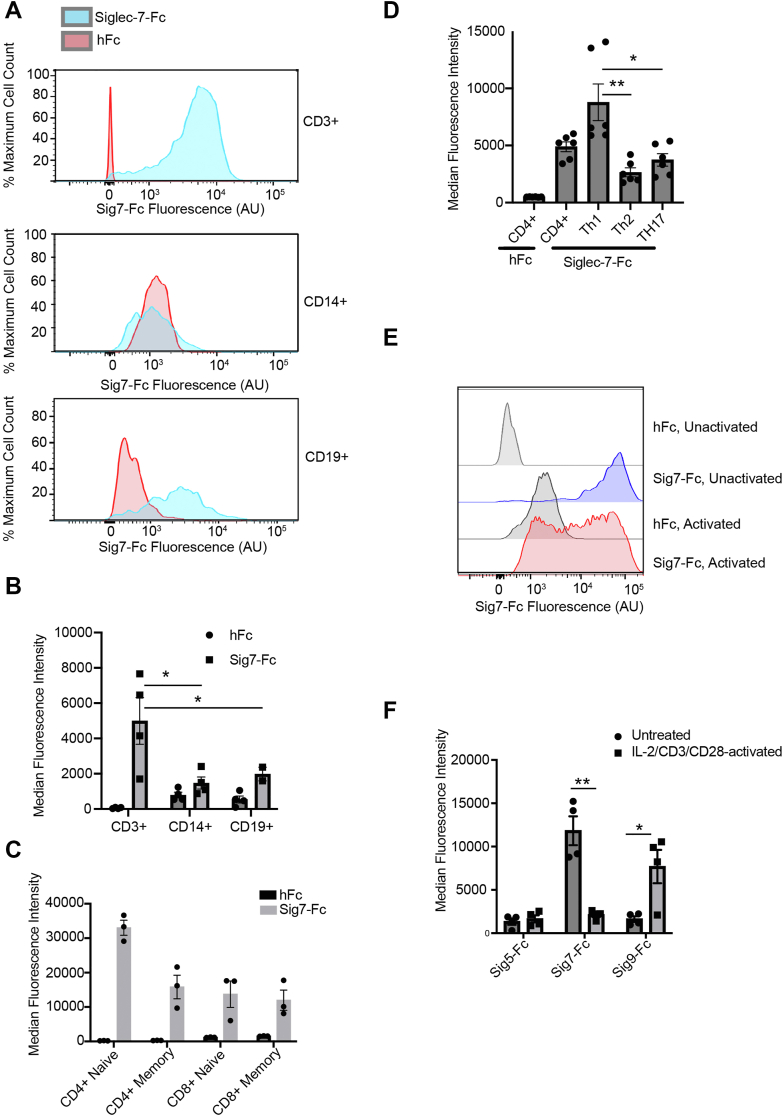
**Siglec-7 ligands are expressed at high levels on primary T-cells.***A*, human PBMCs were stained with 1 μg/ml Siglec-Fc reagents precomplexed to 1 μg/ml AlexFluor488 anti-huFc. Costaining was performed with an anti-CD3-PE-antibody, an anti-CD14-PE antibody, or an anti-CD19 antibody at 1:100 dilution for 30 min. Cells were then analyzed by flow cytometry. Representative flow cytometry plots are shown. *B*, PBMCs were stained and analyzed as in *A*. Average median fluorescence intensity (MFI) for n = 4 donors is plotted. *C*, PBMCs were stained and analyzed as in *A* using Sig7-Fc and antibodies against CD4, CD8, and CD45RO. Average MFI for n = 3 donors is plotted. *D*, PBMCs were stained and analyzed as in *A* using Sig7-Fc and antibodies against CD4, CXCR3, CCR4, and CCR6. Average MFI for n = 6 donors is plotted. *E*, PBMCs were stimulated with anti-CD3 and anti-CD28 antibodies (1 μg/ml) and 0.1 ng/ml IL-2 for 5 days to expand T-cells. Cells were then stained with Siglec-Fcs and CD3-APC as in *A*. Representative flow cytometry plots are shown. *F*, cells were stained as in *E*. Average median fluorescence intensity (MFI) for n = 3 donors is plotted. Statistical significance was determined using a Student’s two-tailed *t-*test, where indicated. ∗ indicates *p* < 0.05, ∗∗ indicates *p* < 0.01. Representative gating strategies are available in Figs. S1 and S2. IL, interleukin; PBMC, peripheral blood mononuclear cell; Sig7-Fc, Siglec-7-Fc; Siglec, sialic acid–binding immunoglobulin-like lectin 7.

We first explored whether Siglec-7-binding glycans are expressed on peripheral immune cells. We sourced peripheral blood mononuclear cells (PBMCs) from several human blood donors and stained them with a recombinant Siglec-7-Fc (Sig7-Fc) protein precomplexed to a fluorescently labeled secondary antibody. This approach is commonly used in the field to detect Siglec ligand expression on cultured cells (2, 32). We also costained with antibodies against the T-cell marker CD3, the monocyte marker CD14, and the B-cell marker CD19. Example of gating strategies for both this and subsequent analyses are given in Figs. S1 and S2. Siglec-7 ligands were uniformly expressed at high levels on CD3+ cells, whereas CD3− cells displayed significantly less staining (Figs. 2, *A* and *B* and S1). While some heterogeneity in staining was observed, nearly all CD3+ cells were positive for Siglec-7 ligands (when compared with a human Fc control). Conversely, CD14+ and CD19+ cells displayed only partial and weak staining with Sig7-Fc. These data confirm that peripheral T-cells express specific cell-surface glycans that bind to the Siglec-7 receptor.

We next investigated the expression of Siglec-7 ligands on both CD4+ and CD8+ T-cell populations as well as on memory (CD45RO+) and naïve (CD45RO−) T-cells (Fig. 2*C*). Particularly high Siglec-7 ligand expression was observed on naïve CD4+ T-cells compared with other T-cell subsets (Fig. 2*C*). However, all T-cell populations expressed Siglec-7 ligands at significantly higher levels than other immune cell types like monocytes or B cells. We also evaluated Sig7-Fc binding to various CD4+ memory T-cell populations, including TH1 (CXCR3+), TH2 (CCR4+CCR6−), and TH17 (CCR4+CCR6+) cells. Sig7-Fc exhibited higher binding to TH1 cells when compared with TH2 cells or TH17 cells, although all cell types displayed significant positivity (Figs. 2*D* and S2). Taken together, these data demonstrate that Siglec-7 ligands are broadly expressed on a range of T-cell subsets. They also hint at interesting subset-specific differences in T-cell glycosylation.

Significant changes in cellular glycosylation have been reported to occur transiently during T-cell activation (33, 34). We therefore also wondered whether T-cells may exhibit altered expression of Siglec ligands following exogenous stimulation. To test this hypothesis, we activated T-cells with agonist antibodies against CD3 and CD28 along with interleukin 2 (IL-2). We then stained cells with a panel of Siglec-Fc reagents 5 days after activation. Interestingly, T-cells showed a dramatic decrease in expression of Siglec-7 ligands after stimulation (Fig. 2, *E* and *F*). This result does not reflect a general decrease in sialic acid expression, as ligands for the related receptor Siglec-9-Fc increased significantly. Taken together, these data confirm that Siglec-7 binds to glycan ligands that are primarily expressed on T-cells and that biosynthesis of these ligands seems to be regulated in a cell type–dependent manner.

### Mucin-type O-glycoproteins bearing disialyl T O-glycans are the major ligands for Siglec-7 on immune cells

**Figure 3:**
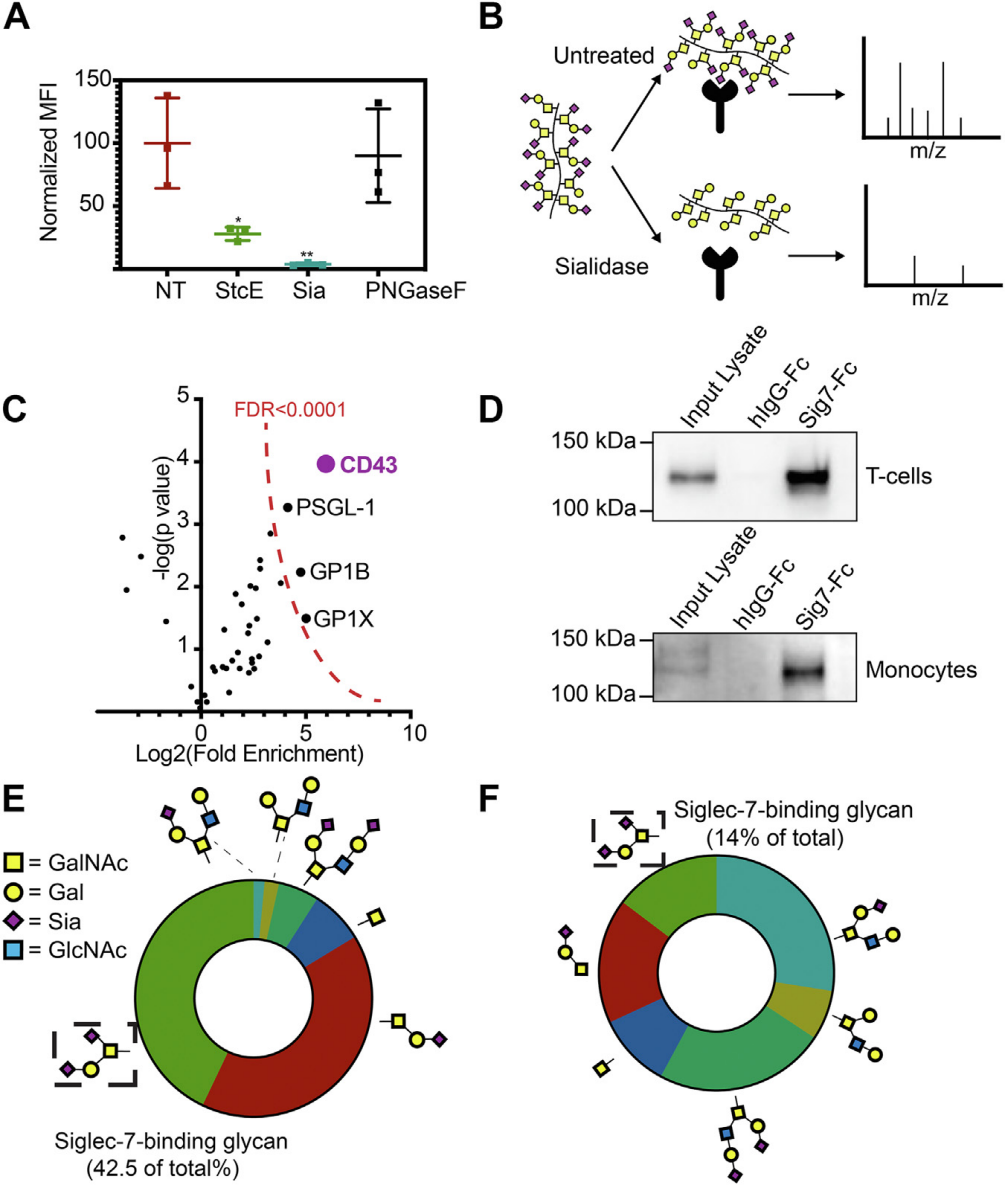
**Disialylcore 1 O-glycans on mucin-type O-glycoproteins are the predominant T-cell ligands for Siglec-7.***A*, human PBMCs were treated with the O-glycoprotease StcE (1 μg/ml), VC-sialidase (Sia, 100 nM), or PNGase F (1:10 dilution) for 30 min at 37 °C. They were then incubated with 1 μg/ml Siglec-7–Fc precomplexed to an AlexaFluor488 anti-huIgG secondary antibody and subjected to live cell flow cytometry. Median fluorescence intensity of all samples was normalized to untreated controls. *B*, workflow of Siglec-7 interactomics analysis. PBMC cell lysates were either left untreated or treated with 100 nM VC-sialidase for 1 h and then incubated with magnetic beads functionalized with recombinant Siglec7-Fc. Tryptic digestion and MS/MS-based identification of Siglec-7-binding proteins was then performed, and the intensity (area under the curve) for each interacting protein was calculated. *C*, enrichment of proteins in untreated *versus* sialidase-treated samples was calculated. Highly enriched proteins (CD43 as well as PSGL-1/GP1B/GPIX) are indicated. Cutoff for statistical significance (FDR < 0.0001) is indicated. *D*, T-cell and monocyte lysates were passed over magnetic beads functionalized with recombinant Siglec-7-Fc and subjected to Western blot with an antibody binding CD43. *E*, T-cells were isolated from PBMCs and subjected to cell-surface glycomic analysis through β-elimination and LC–MS. Relative percentages of each O-glycan as a percentage of total O-glycan structures are given. *F*, monocytes were isolated from PBMCs and subjected to cell-surface glycomic analysis as in *E*. FDR, false discovery rate; PBMC, peripheral blood mononuclear cell; VC, *Vibrio cholera*.

We next assessed what types of glycans serve as the main ligands for Siglec-7 on peripheral immune cells. We treated PBMCs with various glycan-degrading enzymes and then stained cells with Sig7-Fc. Treatment with StcE, an O-glycoprotease that degrades cell-surface O-glycoproteins (35), significantly depleted expression of Siglec-7 ligands. The effect size was similar to that seen from treatment with a sialidase enzyme, which cleaves all cell-surface sialoglycans (Fig. 3*A*). Conversely, *in situ* treatment with PNGase F (which degrades N-linked glycans) had no effect on Sig7-Fc binding (Fig. 3*A*). These data indicate that O-linked glycoproteins are the key ligands for Siglec-7 on peripheral immune cells.

Then, we used a previously described pulldown approach to identify the specific O-linked glycoproteins on PBMCs that bind to Siglec-7 (Fig. 3*B*) (18, 19). PBMCs were isolated, lysed, and incubated with Sig7-Fc complexed to Protein G Dynabeads. Tryptic digestion and LC–MS/MS was then performed to identify bead-bound proteins. To identify glycan-specific binders, some lysates were also treated with sialidase before pulldown. Proteins that bound to beads in untreated samples but not in sialidase treated-controls (*i.e.*, those with significant fold changes with no treatment *versus* sialidase treatment) were identified and ranked. CD43 was the top interacting protein identified by this approach, showing >50-fold enrichment relative to sialidase-treated controls and fourfold more enrichment than any other protein (Fig. 3*C* and Table S1). We also identified a few other mucin-type glycoproteins that interacted with Siglec-7, including PSGL1 and GP1⍺. Interestingly, PSGL-1 has also been identified as a Siglec-7 ligand on B-cell lymphoma cells in a recent study (22). These data underscore that several glycoprotein scaffolds may carry Siglec-7-binding glycans and suggest that CD43 is the most significant of these proteins on human PBMCs.

We previously reported that in K-562 cells, Siglec-7 binds strongly to a specific glycoform of CD43 that is decorated with disialyl core 1 O-glycans (18). This glycoform runs at a molecular weight (MW) of 125 kDa by SDS-PAGE. To confirm that CD43 bears this same glycosylation pattern in human T-cells, we pulled down proteins from T-cell lysates with Sig7-Fc and visualized CD43 by Western blot (Fig. 3*D*). T-cells expressed a single CD43 glycoform that ran at 125 kDa. Conversely, monocytes expressed two distinct glycoforms of CD43: a 125 kDa band that interacted with Siglec-7 and a 150 kDa glycoform that did not (Fig. 3*D*). To independently confirm that this CD43 glycoform is in fact glycosylated with disialyl core 1, we next performed comprehensive glycomics analysis of T-cell O-linked carbohydrates by mass spectrometry (MS). These experiments showed that the disialyl core 1 structure is the most abundant O-glycan on the T-cell surface (Figs. 3*E* and S3). We similarly performed LC–MS analysis of monocyte O-glycans. These experiments showed that monocytes also express some disialyl core 1 structures (Figs. 3*F* and S4). However, a significant fraction of monocyte O-linked carbohydrates was sialylated core 2 glycans, a structure not observed to an appreciable extent on T-cells. Taken together, these findings confirm that Siglec-7 binds to distinct glycoforms of cell-surface O-glycoproteins on the T-cell surface. Notably, they also neatly explain the differences in Siglec-7 ligand expression that we observed between different immune cell subsets (Fig. 2*A*).

### Expression of Siglec-7 ligands on different immune cell subsets is partially regulated by a genetic switch involving the glycosyltransferase GCNT1

**Figure 4:**
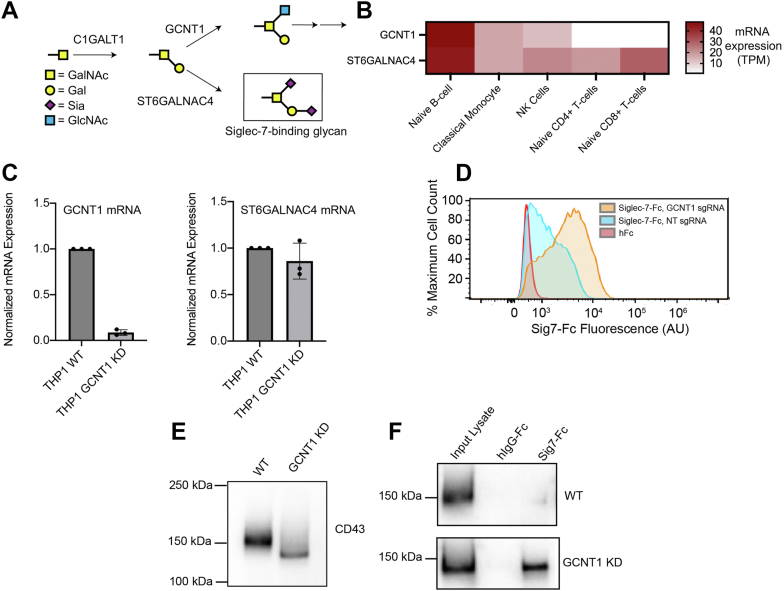
**Biosynthesis of Siglec-7 ligands is regulated in different immune cell subsets by the glycosyltransferase GCNT1.***A*, biosynthesis of different O-glycans in immune cells. The core 1 glycan can be modified either by GCNT1 or ST6GALNAC4 to produce sialylated core 2 and core 1 glycan structures, respectively. *B*, mRNA expression levels (transcript per million [TPM]) for GCNT1 and ST6GALNAC4 in different immune cell subsets. Data sourced from Human Protein Atlas, which has RNA-Seq data for n = 91 healthy donors. *C*, THP-1-dCas9KRAB cells were lentivirally infected with an sgRNA targeting GCNT1, lysed and subjected to RT–quantitative PCR analysis with primers against GCNT1 and ST6GALNAC4. *D*, THP-1 WT and GCNT1 KD cells were stained with 1 μg/ml Sig7-Fc reagents precomplexed to 1 μg/ml AlexFluor488 anti-huFc as described previously and analyzed by flow cytometry. Flow cytometry plot is representative of three independent experiments. *E*, THP-1 WT and GCNT1 KD cells were lysed and subjected to Western blot with an antibody against CD43. *F*, THP-1 WT and GCNT1 KD cells were lysed. Lysate was passed over magnetic beads functionalized with recombinant Sig7-Fc and subjected to Western blot with an antibody binding CD43. Sig7-Fc, Siglec-7-Fc; sgRNA, single guide RNA.

Next, we wanted to understand how expression of Siglec-7 ligands is regulated across different immune cell subsets. Our prior results suggested that expression of Siglec-7 ligands is dependent on the relative synthesis of disialyl core 1 (Siglec-7-binding) and disialyl core 2 (non–Siglec-7-binding) O-glycans. In immune cells, these structures are synthesized by the ST6GALNAC4 and GCNT1 glycosyltransferases, respectively (16). These enzymes compete to add either a GlcNAc (GCNT1) or a sialic acid (ST6GALNAC4) to the 6′-OH group of GalNAc in the core 1 structure (Fig. 4*A*). Given the differences in Siglec-7 ligand expression we observed in T-cells *versus* monocytes (Fig. 2), we wondered whether the relative activity of GCNT1 and ST6GALNAC4 might be different across different immune cell subsets.

We analyzed publicly available RNA-Seq data (Human Protein Atlas, Monaco dataset) from healthy donor PBMCs to determine the mRNA expression levels of both GCNT1 and ST6GALNAC4 in monocytes, NK cells, T-cells, and B cells (36). No major differences in expression of ST6GALNAC4 were observed across these immune subsets. However, GCNT1 expression was significantly repressed in naïve T-cells relative to other cell types (Fig. 4*B*) (36). This finding agrees perfectly with our prior results showing higher expression of disialyl core 2 glycans in monocytes. We next directly tested whether Siglec-7 ligand biosynthesis is altered by changes in the expression of GCNT1. We stably knocked down GCNT1 in THP-1 cells, a leukemia cell line that is frequently used as a cell line model for human monocytes and macrophages (37). THP-1 WT cells displayed relatively low expression of Siglec-7 ligands, mimicking the patterns we observed on primary monocytes. Knockdown of GCNT1, however, significantly increased Siglec-7 ligand expression (Fig. 4, *C* and *D*) (37).

We also used this system to assess how GCNT1 knockdown specifically affects the interaction of Siglec-7 with its primary immune cell ligand CD43. To do this, we lysed THP-1 cells and analyzed expression of CD43 by Western blot. In WT cells, CD43 ran at a 150 kDa MW that is characteristic of monocyte-type glycosylation. Silencing of GCNT1, however, caused CD43 to run at a lower MW similar to that seen in primary T-cells (Fig. 4*E*). Pulldown experiments also showed that Siglec-7 did not interact with CD43 in WT cells. GCNT1 knockdown, however, produced a strong interaction between Siglec-7 and CD43 (Fig. 4*F*). These data demonstrate that Siglec-7 binds selectively to ligands on immune cells decorated with sialylated core 1 but not siaylated core 2 O-linked glycans. They also implicate GCNT1 expression as one key molecular switch that influences the overall production of Siglec-7 ligands in different cell types.

Several previous studies have reported that GCNT1 and core 2 O-glycans are upregulated on primary T-cells following activation with anti-CD3 and anti-CD28 antibodies (38, 39, 40). Our own data showed that Siglec-7 ligand expression is transiently reduced on activated T-cells. We thus wondered whether transcriptional activation of GCNT1 was also responsible for this phenomenon. We did observe that CD43 showed a strong shift in MW by SDS-PAGE following activation of T-cells, indicating that T-cell stimulation does indeed initiate changes in O-glycan biosynthesis (Fig. S5). However, we observed no significant changes in expression of GCNT1 mRNA in activated T-cells by RT–quantitative PCR (qPCR) (Fig. S6*A*). Analysis of publicly available RNA-Seq data (Human Protein Atlas, Schmiedel dataset) similarly showed no change in GCNT1 expression upon stimulation (Fig. S6*B*) (41). Unsurprisingly, these data indicate that Siglec-7 ligand expression is subject to multiple complex layers of regulation beyond that mediated by GCNT1.

### Siglec-7 binds T-cell ligands to regulate T-cell activation and polarization

The Siglec-7 receptor is known to be expressed by antigen-presenting cells (APCs) like dendritic cells (DCs) and macrophages (2, 42). Our work up to this point had shown that Siglec-7 ligands are highly expressed on T-cells. We therefore wondered whether Siglec-7 may regulate T-cell activation by APCs. We optimized a mixed-leukocyte reaction model to test this hypothesis (42). Human monocytes were isolated from PBMCs and differentiated into DCs (42, 43). These cells were found to strongly express the Siglec-7 receptor after 7 days of differentiation (Fig. S7). T-cells from a different donor were then isolated and cocultured with DCs (42). In this assay, allogeneic major histocompatibility complex molecules presented on DCs trigger T-cell activation *in vitro* (42).

**Figure 5:**
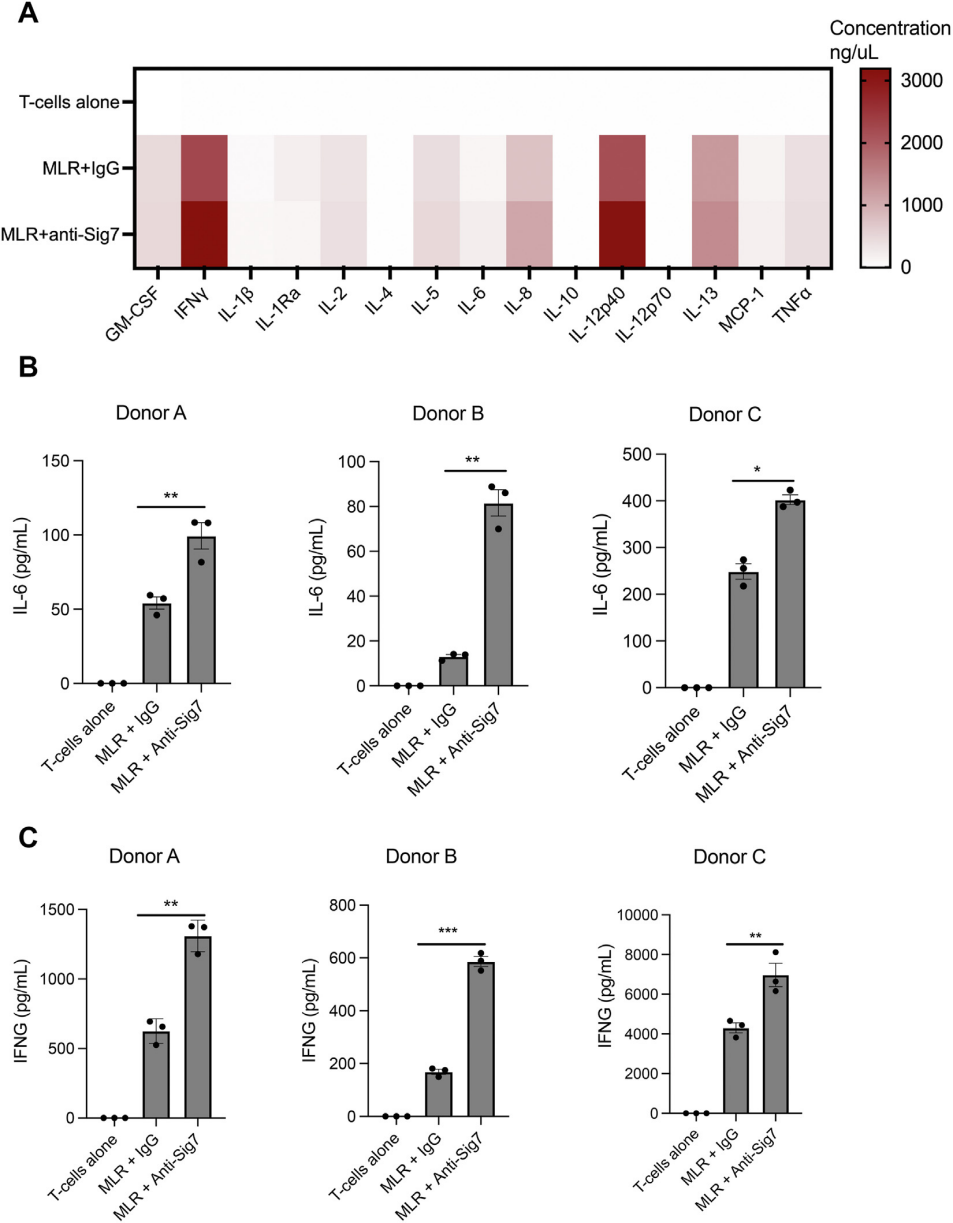
**Blockade of Siglec-7 stimulates T-cell activation by dendritic cells*****in vitro.**A*, T-cells were isolated from various healthy donors and mixed in equal cell ratios with dendritic cells derived from an allogeneic donor. Cells were treated either with Siglec-7 blocking antibody (anti-Sig7) or with an isotype control (20 μg/ml). After 72 h, supernatants were isolated and subjected to Luminex analysis to quantitate secretion of 14 known inflammatory cytokines. Mean values are plotted in a heat-map format. Values averaged for n = 4 different T-cell donors. *B*, effects of Siglec-7 blockade on IL-6 secretion for three separate T-cell donors, as quantitated by Luminex analysis. N = 3 biological replicates (done on different days) are plotted for each donor, ∗∗ indicates *p* < 0.01, ∗∗∗ indicates *p* < 0.001 by two-tailed *t-*test. *C*, effects of Siglec-7 blockade on IFN-γ secretion for three separate T-cell donors, as quantitated by Luminex analysis. N = 3 biological replicates (done on different days) are plotted for each donor. ∗∗ indicates *p* < 0.01, ∗∗∗ indicates *p* < 0.001 by two-tailed *t*-test. IFN, interferon; IL, interleukin.

During coculture, we treated cells with either an immunoglobulin G (IgG) control or a recently described Siglec-7-blocking antibody (44). Following the mixed-leukocyte reaction, we then isolated supernatants from mixed cultures and subjected them to Luminex analysis to determine whether Siglec-7 blockade altered secretion of inflammatory cytokines. Notably, primary T-cells from our donors did not express the Siglec-7 receptor (Fig. S8), matching reports from prior studies (45). Blocking antibodies can thus be assumed to only affect *trans* interactions between APC Siglec-7 and T-cell ligands. Siglec-7 blockade was found to affect secretion of multiple inflammatory factors (Fig. 5*A*). Notably, we observed significant effects both on myeloid-associated cytokines (*e.g.*, IL-6, Fig. 5*B*) and on T-cell-associated cytokines (*e.g.*, IFNγ, Fig. 5*C*) (46, 47, 48). These data confirm that Siglec-7 engages T-cell ligands to regulate intraimmune signaling.

We then sought to explore the mechanism(s) mediating these effects on immune cell activity. The effect of Siglec-7 blockade on APC cytokine secretion can be clearly rationalized. As Siglec-7 possesses inhibitory ITIM/ITSM domains, it is logical that blocking interactions between Siglec-7 and its ligands would enhance costimulation of APCs. The mechanism by which T-cell signaling is affected, however, was less obvious. One recent study found that the related receptor Siglec-15 can directly trigger inhibitory signaling in T-cells by binding to T-cell ligands (9). Soluble galectins have also previously been shown to directly activate specific signaling pathways within T-cells by clustering cell-surface glycoproteins (49, 50, 51). We thus wondered whether Siglec-7 may induce similar direct effects on T-cell activation and polarization.

**Figure 6:**
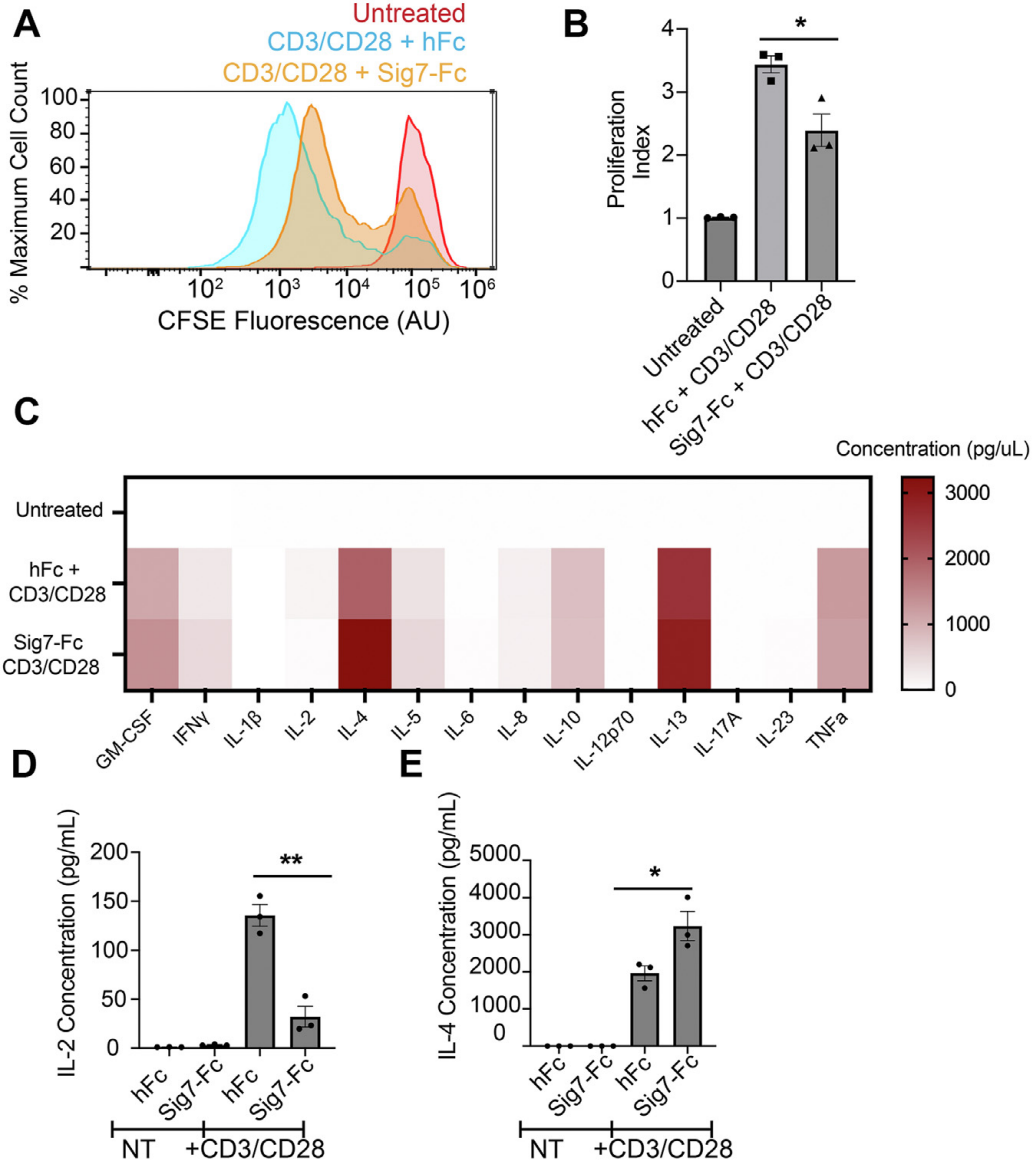
**Siglec-7 directly inhibits the activation of T-cells stimulated*****ex vivo*****.***A*, T-cells were isolated and stimulated with anti-CD3 and anti-CD28 antibodies (1 μg/ml) along with IL-2 (1 ng/ml) for 5 days. T-cells were in addition treated with either rhIgG or Sig7-Fc, along with an anti-huFc secondary antibody to promote ligand crosslinking. Cells were stained with the cell-permeable dye CFSE, and fluorescence was measured by flow cytometry after 5 days. A representative histogram is shown. *B*, cells were treated as in *A*. CFSE plots were analyzed by FlowJo to quantitate a “proliferation index” (total number of divisions divided by number of cells that went into division) for each condition. Results shown are an average of n = 3 T-cell donors. *C*, T-cells were stimulated with anti-CD3 and anti-CD28 antibodies and IgG/Sig7-Fc as in *A*. After 72 h, supernatants were isolated and subjected to Luminex analysis to quantitate cytokine secretion. Mean values for n = 3 biological replicates are plotted in a heat-map format. *D*, T-cells were stimulated as in *C*, and cytokine secretion of IL-2 was quantitated by Luminex analysis. *E*, T-cells were stimulated as in *C*, and cytokine secretion of IL-4 was quantitated by Luminex analysis. Error bars indicate SEM, ∗ indicates *p* < 0.05, ∗∗ indicates *p* < 0.01. CFSE, carboxyfluorescein succinimidyl ester; IL, interleukin; Sig7-Fc, Siglec-7-Fc.

To test this idea, we isolated primary T-cells from healthy donor PBMCs and stimulated them with anti-CD3/anti-CD28 antibodies. We then added either soluble hFc or Sig7-Fc during stimulation. Next, we monitored T-cell proliferation by staining cells with the cell-permeable dye carboxyfluorescein succinimidyl ester (CFSE) and measuring changes in fluorescence over time by flow cytometry. T-cell proliferation results in a gradual reduction of CFSE staining, as the dye is diluted by successive cell divisions (52). Sig7-Fc treatment significantly decreased T-cell proliferation in culture (Fig. 6, *A* and *B*). In parallel, we also quantitated secretion of 14 inflammatory cytokines by Luminex analysis (Fig. 6*C*). Sig7-Fc costimulation similarly induced significant changes in T-cell cytokine secretion. The most significant changes we observed were a reduction in secretion of IL-2 and an increase in secretion of IL-4 (Fig. 6, *D* and *E*). As IL-4 is a classical TH2-polarizing cytokine (53), these results hint that Siglec-7 may play a role in regulating T-cell differentiation into specific functional subsets.

Finally, given these results, we wondered what specific cell-surface Siglec-7 ligand(s) are responsible for transducing these changes in T-cell signaling. Our proteomics results had revealed the CD43 mucin as a primary ligand for Siglec-7 on T-cells. Other glycan-binding proteins like galectins have previously been found to induce changes in T-cell function by binding nonsialylated glycoforms of CD43 (51). We thus hypothesized that Siglec-7 may also affect T-cell function partially through clustering CD43. To assess this idea, we pretreated T-cells with an antibody (MEM-59 clone) that we have previously found to block Siglec-7–CD43 binding (18). We then costimulated T-cells with CD3–CD28–Sig7-Fc and measured T-cell proliferation as aforementioned. Interestingly, we observed that this anti-CD43 antibody elicited a reduction in T-cell proliferation that was similar to that observed for Sig7-Fc (Fig. S9). This functional activity has been described elsewhere in the literature and appears to be a general feature of many CD43-binding antibodies (50, 54, 55). Given these confounding effects, it is difficult to conclusively demonstrate that the functional changes we observe from Sig7-Fc costimulation are specifically linked to Sig7-Fc–CD43 binding. However, we do think it is suggestive that both Sig7-Fc and anti-CD43 antibodies seem to bind the same ligand and induce similar functional effects on T-cells. When considered together, all these data convincingly demonstrate a previously unrecognized function for Siglec-7 in regulating T-cell activation and differentiation.

## Discussion

In previous work, we had shown that O-glycoproteins bearing disialyl core 1 O-glycans are the key ligands for Siglec-7 on a number of immortalized cell lines. This work makes a significant advance by demonstrating for the first time that such ligands are also broadly expressed on nontransformed and primary cells. We further demonstrate a physiological function for these ligands in regulation of T-cell activation by dendritic cells. Finally, we confirm that transcriptional activation and/or repression of specific O-glycan biosynthesis enzymes can be a critical biological switch regulating expression of Siglec-7 ligands on the surface of immune cells (19, 22). This work thus adds to a growing weight of evidence that sialylated O-linked glycans are the primary functional ligands for Siglec-7 in a wide range of cell types (16, 18, 22, 56).

In recent years, there have been significant efforts to develop Siglec-blocking agents as cancer immunotherapies (2, 8, 57). Such therapeutics have been presumed to work by blocking inhibitory interactions between immune cell Siglecs and ligands expressed on cancer cells. However, our results imply that Siglec-7 may also regulate priming of T-cells by APCs. Stimulation of T-cell priming is a critical mechanism of action for therapeutics targeting traditional immune checkpoints like PD-1, PD-L1, and CTLA-4 (58, 59, 60). The identification of analogous “glycoimmune” T-cell checkpoints may thus be highly significant for the design and development of novel immunotherapies (2). Going forward, it will be essential to validate this hypothesis more deeply in preclinical immune oncology models and immune cells isolated from human cancer patients. Notably, a recent study reported the development of a humanized mouse model where Siglec-7 was stably expressed in murine immune cells (44). The emergence of these new tools for studying Siglec biology may now allow for more precise dissection of how Siglec-7 regulates T-cell anticancer activity *in vivo*.

We also found that treatment of T-cells with recombinant Siglec-7 induces significant changes in T-cell proliferation and cytokine secretion patterns. These data imply that Siglec-7 may cluster specific cell-surface ligands and directly trigger intracellular signaling cascades within T-cells. This type of noncanonical signaling mechanism is novel for Siglec-7 but has been described for other glycan-binding proteins (49, 50, 51) as well as for the related sialic acid–binding receptor Siglec-15 (9). The specific signaling pathways that are activated within T-cells following cell-surface binding of Siglec-7 are yet to be defined. While signaling through CD43 is one possible mechanism, we would note that Siglec-7 interacts with other O-glycosylated ligands on T-cells, implying that Siglec-7 costimulation may induce complex signaling through multiple pathways. In addition, while Siglec-7 is not expressed on peripheral T-cells, it is sometimes upregulated on T-cell subsets in the tumor microenvironment (7, 61). Other reports have also described attempts to engineer Siglec-based CAR-T cells that target tumor cell sialoglycans (62, 63). *Cis* interactions between Siglec-7 and T-cell ligands could also regulate T-cell activity in these contexts. Comprehensive characterization of these signaling mechanisms is thus an important goal for future work.

Finally, while our studies here have focused mainly on Siglec-7, these findings likely have broader implications for understanding how other immune lectins control adaptive immunity. So far, most prior research on T-cell checkpoints has focused on protein–protein interaction interfaces. However, the central role of glycosylation in regulating development, activation, and polarization of T-cells (recently well reviewed in Ref. (64)) has become ever-clearer in recent years. It is likely that ligands for other members of the Siglec family are expressed on T-cells and modulate this axis of immune regulation. Comprehensive structural and functional characterization of these ligands thus continues to be a rich area for future investigation.

## Experimental procedures

### Isolation of PBMCs

Leukoreduction cones from anonymous and healthy blood donors were sourced either from the Stanford Blood Centre or from STEMCELL Technologies (200-0093). Donor blood was processed within 24 h of the initial blood draw. About 8 to 10 ml of patient blood was diluted to 30 ml total volume in PBS and layered gradually onto 15 ml Ficoll-Paque density gradients (GE Healthcare). Samples were centrifuged at 1100*g* in a table-top swinging bucket centrifuge with the brake off (minimum acceleration/deceleration settings) for 20 min. PBMCs were carefully isolated from the Ficoll–PBS interface in a total of 2 to 3 ml and then diluted directly into 50 ml PBS. Cells were resuspended in 30 ml PBS and centrifuged for 10 min at 600*g*. PBMCs were then frozen in fetal bovine serum (FBS) containing 10% dimethyl sulfoxide overnight at −80 °C in a cryogenic freezing chamber. PBMCs were then transferred to a liquid nitrogen storage unit for long-term storage.

### Cell culture

THP-1 cells were sourced from the American Type Culture Collection and cultured in RPMI medium supplemented with 10% FBS (Thermo Fisher Scientific) at 5% CO_2_ and 37 °C. Following isolation, PBMCs were cultured either in RPMI with 10% FBS or in differentiation medium specific to a given cell type (detailed later).

### T-cell activation and expansion

About 1.2 × 10^7^ thawed PBMCs isolated from n = 4 healthy donor–supplied leukocyte reduction system cones (STEMCELL) were seeded at 2 × 10^6^ cells/ml of ImmunoCult-XF T Cell Expansion Medium (STEMCELL) for a total of 2 × 3 ml in 6-well plates. PBMCs were either untreated (ImmunoCult-XF T Cell Expansion Medium alone) or activated (treated with ImmunoCult-XF T Cell Expansion Medium containing 1 μg/ml CD3, 1 μg/ml CD28, and 100 ng/ml IL-2) for 72 h, after which the cells were collected and reseeded in fresh media as described previously for a further 48 h. Anti-CD3 and anti-CD28 antibodies were sourced from BioXL. IL-2 was from PeproTech.

### Mixed leukocyte reaction

Monocytes were isolated from thawed PBMCs using the EasySep Human Monocyte Isolation Kit (STEMCELL) and plated at 400,000 cells/well in 96-well plates in ImmunoCult-ACF Dendritic Cell Medium. Differentiation was performed using the ImmunoCult Dendritic Cell Culture Kit (STEMCELL) for 7 days according to the manufacturer’s instructions. After 7 days, T-cells from a different donor were isolated using the EasySep Human T-cell Isolation Kit. Expired media were removed, and T-cells were added in fresh media to DCs (100,000 T-cells/well) along with blocking antibodies at the indicated concentrations. After 72 h, supernatants were isolated by two rounds of centrifugation at 1000*g* for 10 min. Luminex analysis was performed at EVE Technologies Corporation.

### Siglec-Fc pulldowns

T-cells and monocytes were isolated from thawed PBMCs using the EasySep Human T-cell Isolation Kit or the EasySep Human Monocyte Isolation Kit. Cells were lysed in ice-cold 0.1% Nonidet P-40 with 1× Halt Protease Inhibitor mixture (Thermo Fisher Scientific) in PBS and rotated at 4 °C for 1 h. Lysates were centrifuged at 18,000*g* in a table-top microcentrifuge for 15 min to remove any insoluble material. The bicinchoninic acid assay was used to determine protein concentration. Lysates were then diluted in lysis buffer to a final concentration of 1 μg/ml. In parallel, either 2.5 μg rhIgG-Fc (R&D Biosystems) or Sig7-Fc (1138-SL-050) was mixed with 25 μl Protein G Dynabeads (Thermo Fisher Scientific) in 250 μl PBS and rotated at room temperature for 1 h. Beads were isolated with a magnet and washed once with PBS. Cell lysates were then added to Siglec-coated beads and rotated at 4 °C overnight. The following day, beads were washed three times with 200 μl of cold lysis buffer. Bound protein was eluted by boiling beads in 1× SDS-PAGE sample buffer with 2-mercaptoethanol for 5 min prior to loading onto a 4 to 12% Tris–glycine gel. Western blotting was performed with an anti-CD43 antibody (MEM-59 clone; Thermo Fisher Scientific).

### Siglec-Fc staining for flow cytometry

Siglec-Fc precomplexes were prepared by diluting both Siglec-Fc (R&D Biosystems) and anti-huIgG–Alexa Fluor 488 (Jackson ImmunoResearch; catalog no.: 109-545-008) to 1 μg/ml in PBS for 1 h in ice. Cells were then resuspended in precomplex solution at the indicated cell densities and incubated for 30 min on ice in 96-well V-bottom plates. Cells were subsequently centrifuged and washed with cold PBS and analyzed by flow cytometry on either a BD Accuri C6, a BD LSR II Analyzer, or a CytoFlex benchtop flow cytometer. Gating on forward scatter/side scatter was used to identify intact cells. FSC-A *versus* FSC-H gating was used to eliminate doublet cells. Antibody staining was performed with a 1:200 dilution of the indicated antibodies (sourced from BioLegend) for 30 min. Where indicated, PBMCs were sometimes pretreated prior to Siglec-Fc staining with either 1 μg/ml of the O-glycoprotein-specific protease StcE or 100 nM of *Vibrio cholerae* sialidase for 1 h.

### Generation of GCNT1 KD line

THP-dCas9KRAB cells were generated and lentivirally infected with a single guide RNA (sgRNA) against GCNT1 using a previously described protocol (18). The sgRNA sequence 5′GTCTAAGCTACACGGACCAG3′ was chosen based on the CRISPRi sgRNA library sequences given (65).

### MS-based glycomics

Either T-cells or monocytes were isolated using bead-based isolation kits (STEMCELL, see aforementioned) and lysed in high salt buffer using probe sonication. Lysed cells were centrifuged, and the supernatant was stored. The cell pellets of samples were then dissolved in urea lysis buffer, reduced using DTT, and alkylated using iodoacetamide. The samples were then dialyzed against the water at 4 °C for 48 h to remove urea. Water was changed every 4 to 6 h. The sample solutions were diluted by adding 10× PNGAse F glycobuffer to make it 1× and again sonicated to dissolve the proteins. The released N-glycans were filtered off using 10 KDa cutoff filter and purified using a c18 cartridge. O-glycoproteins from top of the filter were subjected to β-elimination. The O-glycoproteins were treated with a mixture of 50 mM NaOH solution and sodium borohydride (NaBH_4_) solution in 50 mM NaOH solution. The samples were heated to 45 °C for 18 h. The samples were cooled, neutralized by 10% acetic acid, passed through Dowex H^+^ resin column and c18, lyophilized, and borates were removed under the stream of nitrogen using methanol and acetic acid mixture. The dried eluate was dissolved with dimethyl sulfoxide and methylated by using methyl iodide on dimethyl sulfoxide–NaOH mixture. The reaction was quenched with water, and the reaction mixture was extracted with methylene chloride and dried. The dried glycans were redissolved in methanol and profiled by MALDI-TOF. N- and O-glycans were redissolved in 20 μl methanol. About 5 μl of each sample was air dried to approximately 1 μl. About 1 μl 2,5-dihydroxybenzoic acid matrix was added and spotted on MALDI plate. The samples were analyzed on AB Sciex TOF/TOF 5800 System Mass Spectrometer using reflector positive ion mode.

### T-cell proliferation monitoring

About 3.5 × 10^7^ thawed PBMCs were spun down and washed with PBS. The PBMCs were stained with a 5 μM CFSE Working Solution according to the manufacturer’s protocol (BioLegend; catalog no.: 423801). After staining, they were then washed with PBS and brought up to 500,000 cells/ml in ImmunoCult-XF T Cell Expansion Medium. The PBMCs were then plated at 100,000 cells per well in a 96-well plate. The PBMCs were either untreated (ImmunoCult-XF T Cell Expansion Medium alone), activated (treated with ImmunoCult-XF T Cell Expansion Medium containing 1 μg/ml CD3, 1 μg/ml CD28, and 0.1 ng/ml IL-2), or activated and treated with anti-huFc (1 μg/ml) and either Sig7-Fc (1 μg/ml) or IgG-Fc (1 μg/ml) for 5 days after which the cells were collected and stained with CD3-PE for flow analysis.

### Siglec immunoprecipitation and proteomics sample preparation

PBMC pellets (at least 50 × 10^6^ cells) were resuspended in lysis buffer (1 × PBS + 0.1% NP-40 [Abcam] + 1 × Halt protease inhibitor cocktail [Thermo Fisher Scientific]) and lysed *via* sonication. Lysates were clarified *via* centrifugation at 20,000*g* for 10 min at 4 °C, and lysate concentration was normalized to 1 mg ml^−1^ total protein in lysis buffer. Immunoprecipitation samples were prepared in parallel with untreated lysate and lysate pretreated with 100 nM *V. cholerae* sialidase for 3 h at 37 °C. During sialidase treatment, Siglec-Fc (R&D Biosystems) was precomplexed on Protein G beads (Invitrogen). For each sample, 50 μl Protein G beads were aliquoted into a 1.5 ml microcentrifuge tube and washed once with 250 μl PBS. Siglec-Fc was added to beads (250 μl of 20 μg ml^−1^ Siglec-Fc stock in 1× PBS) and precomplexed *via* incubation for 1 h at room temperature with rotation. After 1 h, beads were washed once with 250 μl PBS to remove unbound Siglec-Fc. Following sialidase treatment, 500 μl sialidase treated or untreated lysate was added to the precomplexed Siglec-Fc magnetic beads and incubated overnight at 4 °C with rotation. Immunoprecipitations were performed in biological triplicate.

For Western blot analysis, beads were boiled in SDS-PAGE buffer, loaded on a 4% to 12% Tris-Glycine Gel (BioRad) and subsequently analyzed. For mass spectrometry, beads were washed twice with 200 μl lysis buffer and three times with 200 μl MS grade 50 mM triethylammonium bicarbonate (TEAB; Thermo Fisher Scientific). Immunoprecipitated ligands were eluted from beads by boiling in 50 μl 50 mM TEAB + 0.05% Rapigest (Waters) for 10 min at 100 °C, and eluate was collected. Beads were washed with an additional 50 μl 50 mM TEAB + 0.05% Rapigest, which was combined with the first eluate fraction. Eluates were reduced *via* addition of 5 mM DTT (Sigma) and incubation at 60 °C for 30 min with shaking. Samples were then alkylated *via* addition of 10 mM iodoacetamide (Sigma) and incubation at room temperature for 30 min with shaking. Samples were further digested with 1 μg sequencing grade trypsin (Promega) *via* overnight incubation at 37 °C with shaking. Samples were acidified *via* addition of MS grade 2% formic acid (FA; Sigma) and incubation at 37 °C for 30 min with shaking. Samples were then dried in a vacuum concentrator and cleaned up using Strata-X columns (Phenomenex). Strata-X columns were activated with 1 ml MS grade acetonitrile (ACN; Sigma). Dried samples were then resuspended in 1 ml 0.1% FA in MS grade water (Sigma) and loaded onto activated Strata-X columns. Columns were washed with 1 ml 0.1% FA, and samples were eluted with 400 μl 0.1% FA in 80% ACN in water. Eluates were dried in a vacuum centrifuge and resuspended in 10 μl 0.1% FA. Peptide concentrations were measured *via* A205 on a Nanodrop (Thermo Fisher Scientific) and normalized to 1 mg ml^−1^ in 0.1% FA. Immunoprecipitated ligands were identified *via* LC–MS/MS analysis.

### MS identification of Siglec ligands

For each immunoprecipitation sample, 1 μg was injected for LC–MS/MS analysis. Peptides were separated over a 25 cm EasySpray reverse-phase LC column (75 μm inner diameter packed with 2 μm, 100 Å, PepMap C18 particles; Thermo Fisher Scientific). The mobile phases (A: water with 0.2% FA and B: ACN with 0.2% FA) were driven and controlled by a Dionex Ultimate 3000 RPLC nano system (Thermo Fisher Scientific). An integrated loading pump was used to load peptides onto a trap column (Acclaim PepMap 100 C18, 5 μm particles, 20 mm length; Thermo Fisher Scientific) at 5 μl/min, which was put in line with the analytical column 5 min into the gradient. The gradient was held at 0% B for the first 6 min of the analysis, followed by an increase from 0% to 5% B from 6 to 7 min, and increase from 5 to 25% B from 7 to 66 min, an increase from 25% to 90% from 66 to 70 min, isocratic flow at 90% B from 70 to 75 min, and re-equilibration at 0% B for 15 min for a total analysis time of 90 min. Eluted peptides were analyzed on an Orbitrap Fusion Tribrid MS system (Thermo Fisher Scientific). Precursors were ionized using an EASY-Spray ionization source (Thermo Fisher Scientific) held at +2.2 kV relative to ground, the column was held at 40 °C, and the inlet capillary temperature was held at 275 °C. Survey scans of peptide precursors were collected in the Orbitrap from 350 to 1350 Th with an automatic gain control target of 1,000,000, a maximum injection time of 50 ms, RF lens at 60%, and a resolution of 60,000 at 200 *m/z*. Monoisotopic precursor selection was enabled for peptide isotopic distributions, and precursors of z = 2 to 5 were selected for data-dependent MS/MS scans for 2 s of cycle time. Dynamic exclusion was set to exclude precursors after being selected once for an exclusion time of 30 s with a ±10 ppm window set around the precursor monoisotope. An isolation window of 1 Th was used to select precursor ions with the quadrupole, and precursors were fragmented using a normalized higher-energy collisional dissociation energy of 30. MS/MS scans were collected with an automatic gain control target of 100,000 ions, with a maximum accumulation time of 54 ms and an Orbitrap resolution of 30,000 at 200 *m/z*. The same method was used for both untreated and sialidase-treated samples. Raw data were processed with MaxQuant (66), version 1.6.2.10, and tandem mass spectra were searched with the Andromeda search algorithm (67). About 20, 4.5, and 20 ppm were used for first search MS1 tolerance, main search MS1 tolerance, and MS2 product ion tolerance, respectively. Oxidized methionine and deamidated asparagine were set as variable modifications, and carbamidomethylation of cysteine was set as a fixed modification. Cleavage specificity was set to Trypsin/P with two missed cleavage allowed. Peptide spectral matches were made against a human protein database (reviewed entries only, 20,416 entries total) downloaded from UniProt. Peptides were filtered to a 1% false discovery rate (FDR) using a target-decoy approach, and a 1% protein FDR was applied (68). Proteins were quantified and normalized using MaxLFQ, and the match between runs feature was enabled. Label-free intensity values were log2 transformed and plotted using Perseus version, 1.6.2.2 (66). Identified proteins were filtered to plot those that are annotated as localized on cell surface or secreted but not cytoplasmic (*via* UniProt annotations). Significance cutoffs for volcano plots were determined using Student’s *t*-test with an FDR of 0.0001 and minimum enrichment (S_0_) of 5.

### Multiplex analysis of cytokines

In this study, we used Luminex xMAP technology for multiplexed quantification of 15 human cytokines, chemokines, and growth factors. The multiplexing analysis was performed using the Luminex 200 system (Luminex) by the Eve Technologies Corporation. Fifteen markers were simultaneously measured in the samples using Eve Technologies' Human Focused 15-Plex Discovery Assay (MilliporeSigma) according to the manufacturer's protocol. The 15-plex consisted of granulocyte–macrophage colony-stimulating factor, IFNγ, IL-1β, IL-1Ra, IL-2, IL-4, IL-5, IL-6, IL-8, IL-10, IL-12p40, IL-12p70, IL-13, monocyte chemoatrractant protein-1, and tumor necrosis factor alpha. Assay sensitivities of these markers range from 0.14 to 5.39 pg/ml for the 13-plex. Individual analyte sensitivity values are available in the MilliporeSigma MILLIPLEX MAP protocol.

### RT–qPCR analysis

RNA was extracted from THP1 wildtype and THP1 GCNT1 KD cells using the Monarch Total RNA Miniprep kit (NEB) following the manufacturer's protocol. Complementary DNA was then generated using the NEB RT SuperMix kit following the manufacturer's protocol. Primers were synthesized by IDT and dissolved in water to a working concentration of 10 μm. NEB Universal qPCR Master mix, forward/reverse primers, water, and complementary DNA were mixed together in a qPCR 96-well plate on ice. A StepOnePlus Real-Time PCR System was set up using the following parameters: 30 s at 95 °C, 15 s at 95 °C followed by 30 s at 60 °C for 35 cycles. Gene expression was calculated using the comparative CT method in which the gene of interest was normalized to expression of hypoxanthine guanine phosphoribosyltransferase.

## Data availability

All data are included in the article and/or supporting information. Any questions regarding data availability can be directed to the corresponding author.

## Supporting information

This article contains supporting information.

## Conflict of interest

C. R. B. is a cofounder and Scientific Advisory Board member of Palleon Pharmaceuticals, Enable Bioscience, Redwood Biosciences (a subsidiary of Catalent), InterVenn Biosciences, Lycia Therapeutics, Virsti Therapeutics, OliLux Biosciences, and GanNA Bio. S. W. and C. R. B. are coinventors on a patent application related to work reviewed in this article held by Stanford University (grant no.: PCT/US2020/041603).
