# Supplementary material for: The glycoimmune checkpoint receptor Siglec-7 interacts with T-cell ligands and regulates T-cell activation
Source: J Biol Chem. 2023 Dec 21;300(2):105579. doi: 10.1016/j.jbc.2023.105579 (PMC10831161; doi:10.1016/j.jbc.2023.105579)
Supplement: Supporting Figures S1–S9 [file mmc1.docx]

`­­

**Supplementary Information for:**

**The glyco-immune checkpoint receptor Siglec-7 interacts with T-cell ligands and regulates T-cell activation**

Natalie Stewart^1^, John Daly^1^, Olivia Drummond-Guy^1^, Vignesh Krishnamoorthy^1^ Jessica C. Stark^2^, Nicholas M. Riley^4^, Karla C. Williams^1^, Carolyn R. Bertozzi^2,3^, Simon Wisnovsky^1^**^,*^**.

^1^University of British Columbia, Faculty of Pharmaceutical Sciences, Vancouver, V6T 1Z3.

^2^Department of Chemistry & Sarafan ChEM-H, Stanford University, Stanford, California 94305, USA.

^3^Howard Hughes Medical Institute, Stanford, California 94305, USA.

^4^University of Washington, Department of Chemistry, Seattle, WA, 98195, USA.

Corresponding Author: Simon Wisnovsky

simon.wisnovsky@ubc.ca

**This PDF file includes:**

Supplementary Figures


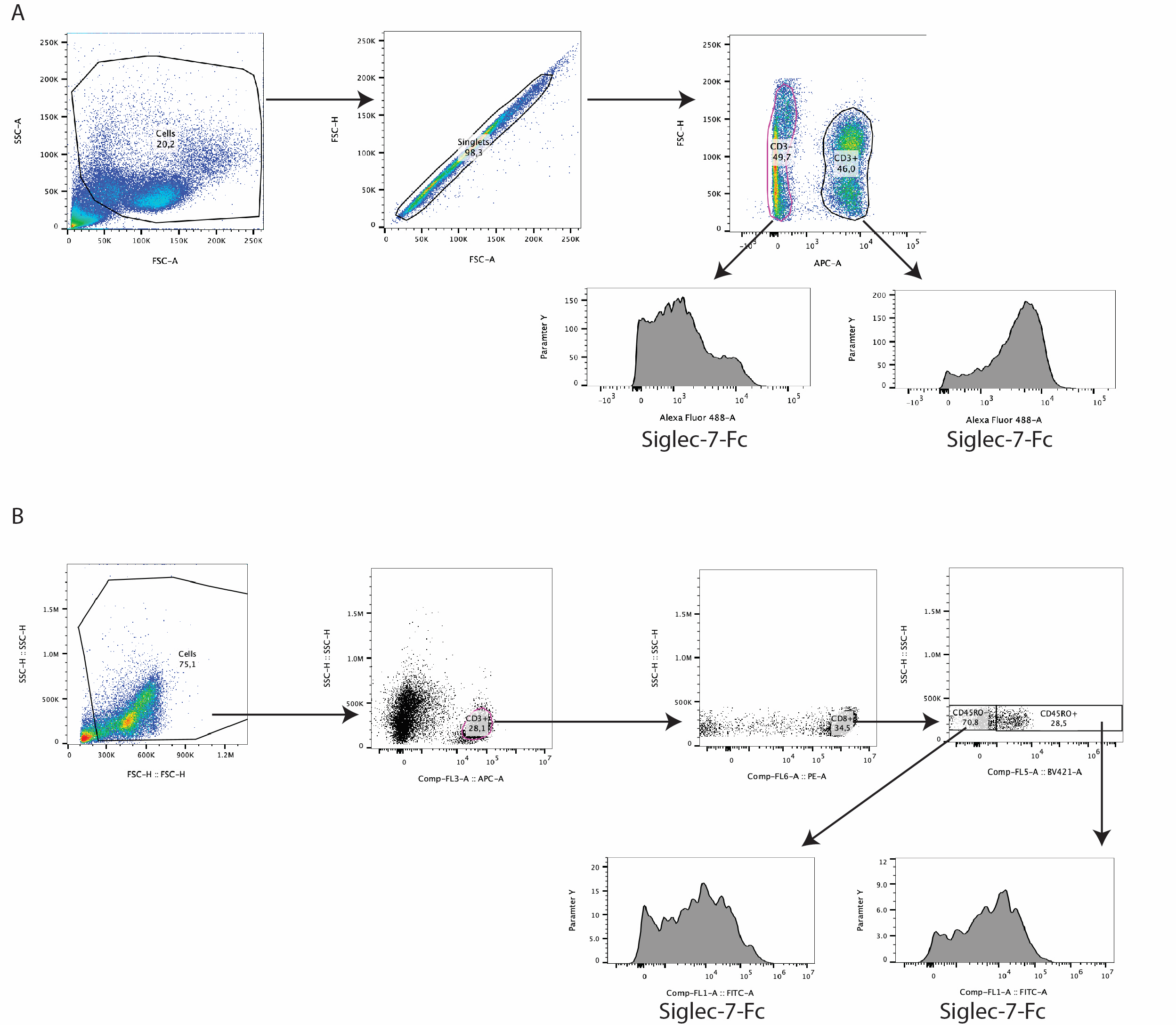


**Supplementary Figure 1. A)** Representative gating strategy for T-cell profiling data presented in Fig. 2A-B **B)** Representative gating strategy for T-cell profiling data presented in Fig. 2C. All gates set based on FMO controls.


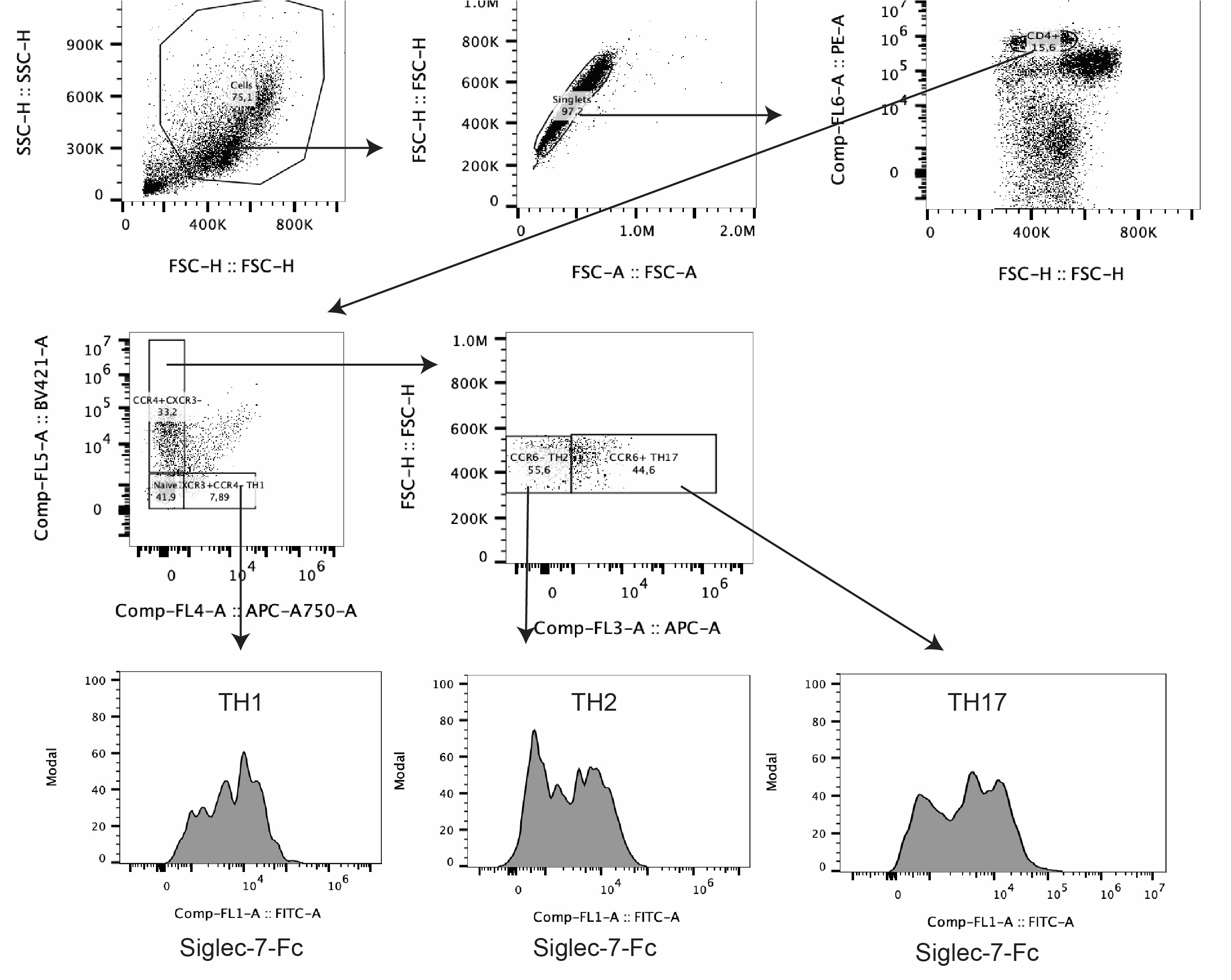


**Supplementary Figure 2.** Representative gating strategy for T-cell profiling data presented in Fig. 2D

**
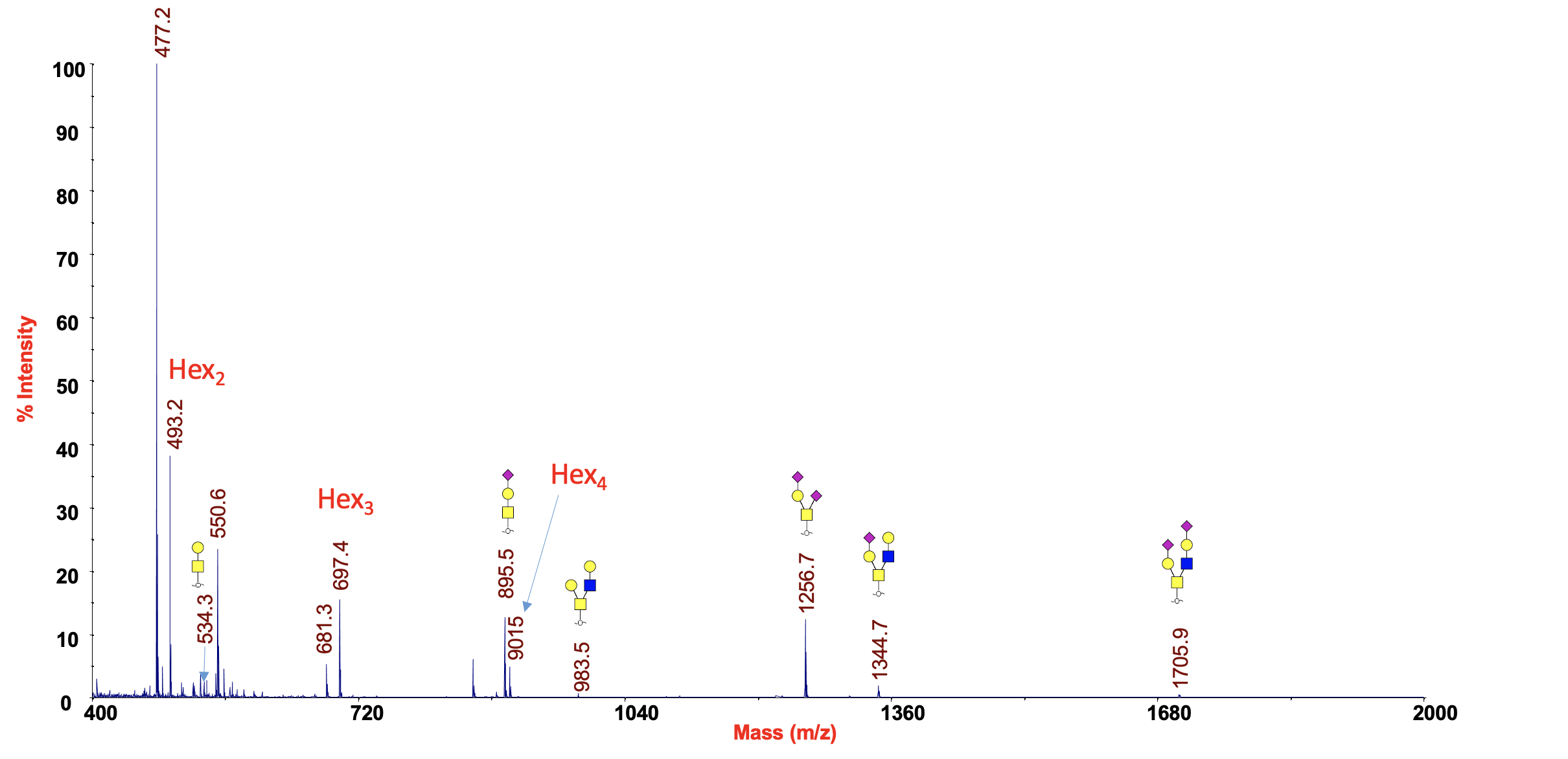
**

**Supplementary Figure 3.** Full MALDI TOF/TOF mass spectrum of released and permethylated O-linked glycans derived from isolated peripheral T-cells.

**
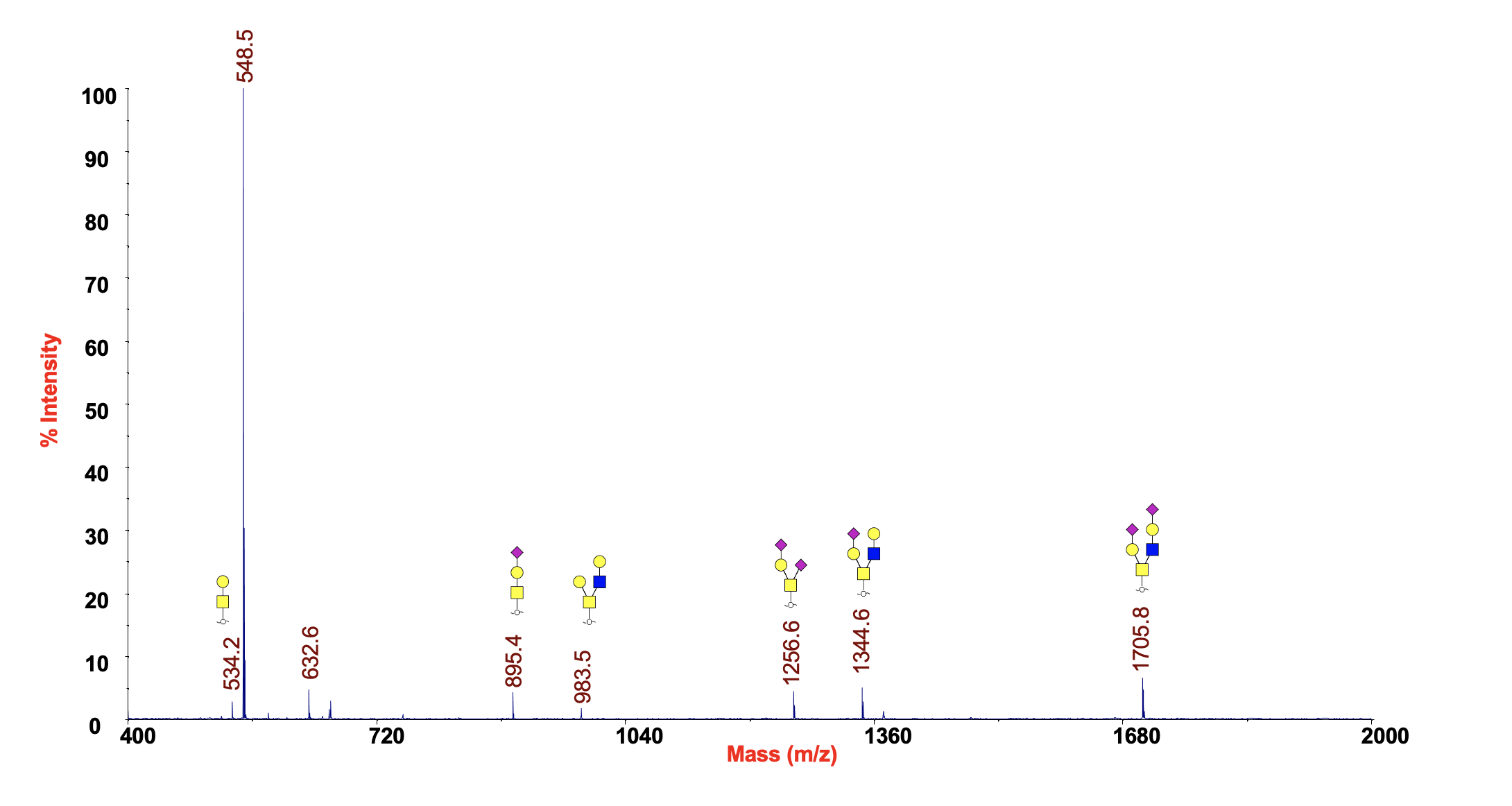
**

**Supplementary Figure 4.** Full MALDI TOF/TOF mass spectrum of released and permethylated O-linked glycans derived from isolated peripheral monocytes.


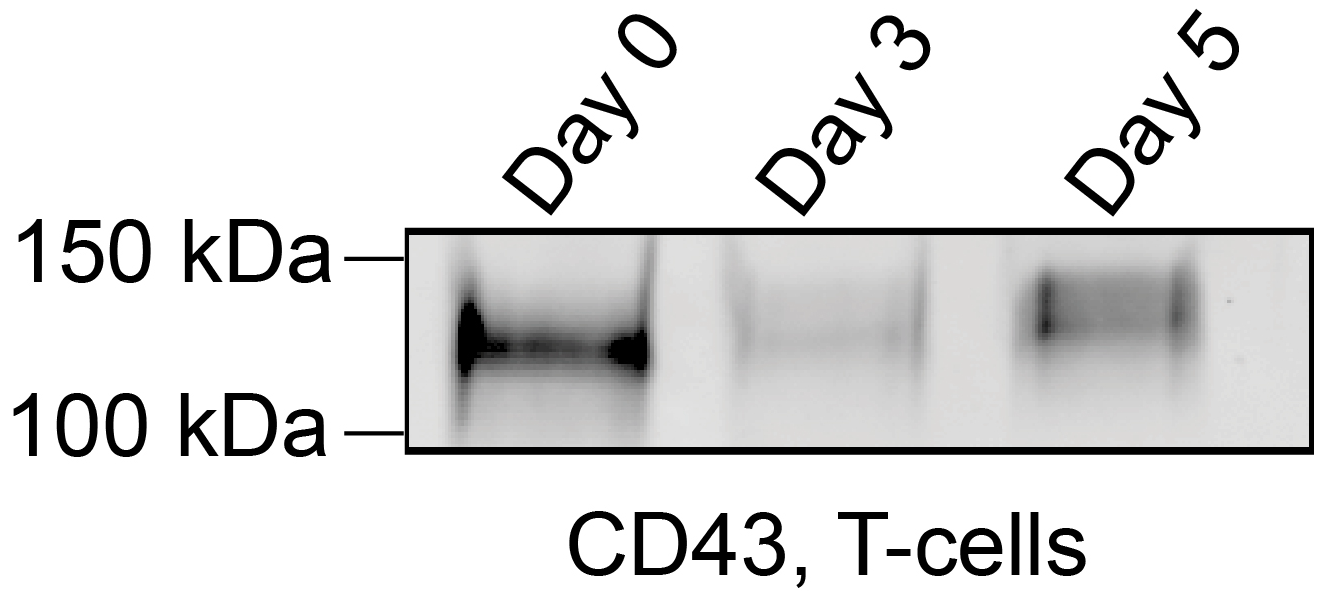


**Supplementary Fig. 5** T-cells were stimulated with CD3/CD28 antibodies and IL-2 for 5 days. Lysate was extracted at the indicated time points and CD43 was detected by immunoblot (MEM-59 clone).


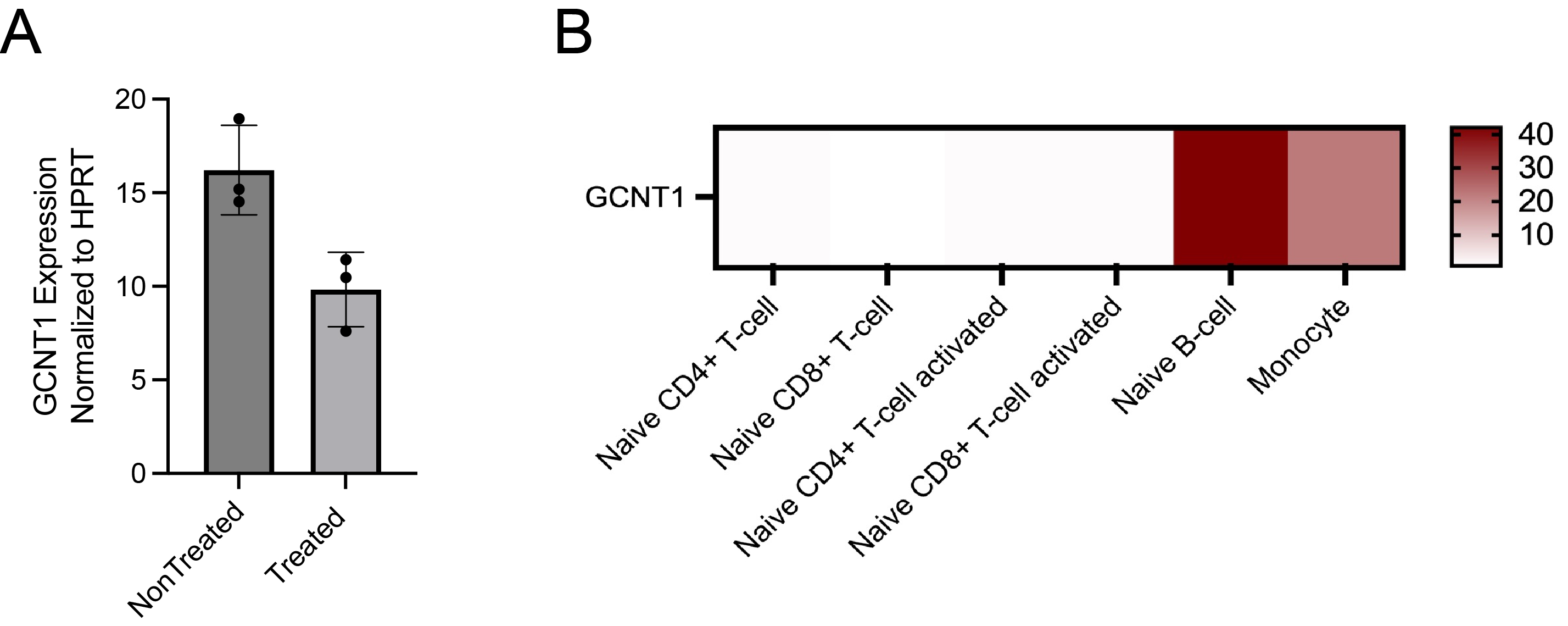


**Supplementary Fig. 6 A)** T-cells were stimulated with CD3/CD28 antibodies and IL-2 for 5 days. RNA was extracted and subjected to RT-qPCR analysis with primers against GCNT1. Gene expression was normalized to the HPRT housekeeping gene **B)** Expression of GCNT1 mRNA (TPM) in T-cells activated for 4 hours with CD3/CD28 antibodies was measured by RNA-seq (data from Human Protein Atlas). B-cell and monocyte expression levels are shown for comparison.

**
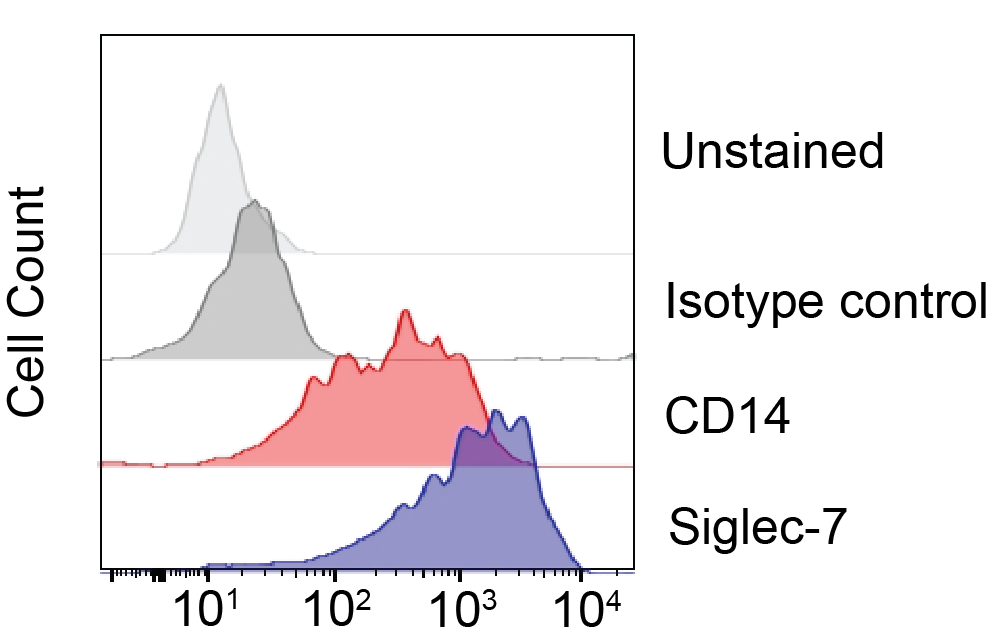
**

**Supplementary Figure 7.** Representative flow cytometry plot showing expression of Siglec-7 on dendritic cells 7 days following isolation and differentiation.

**
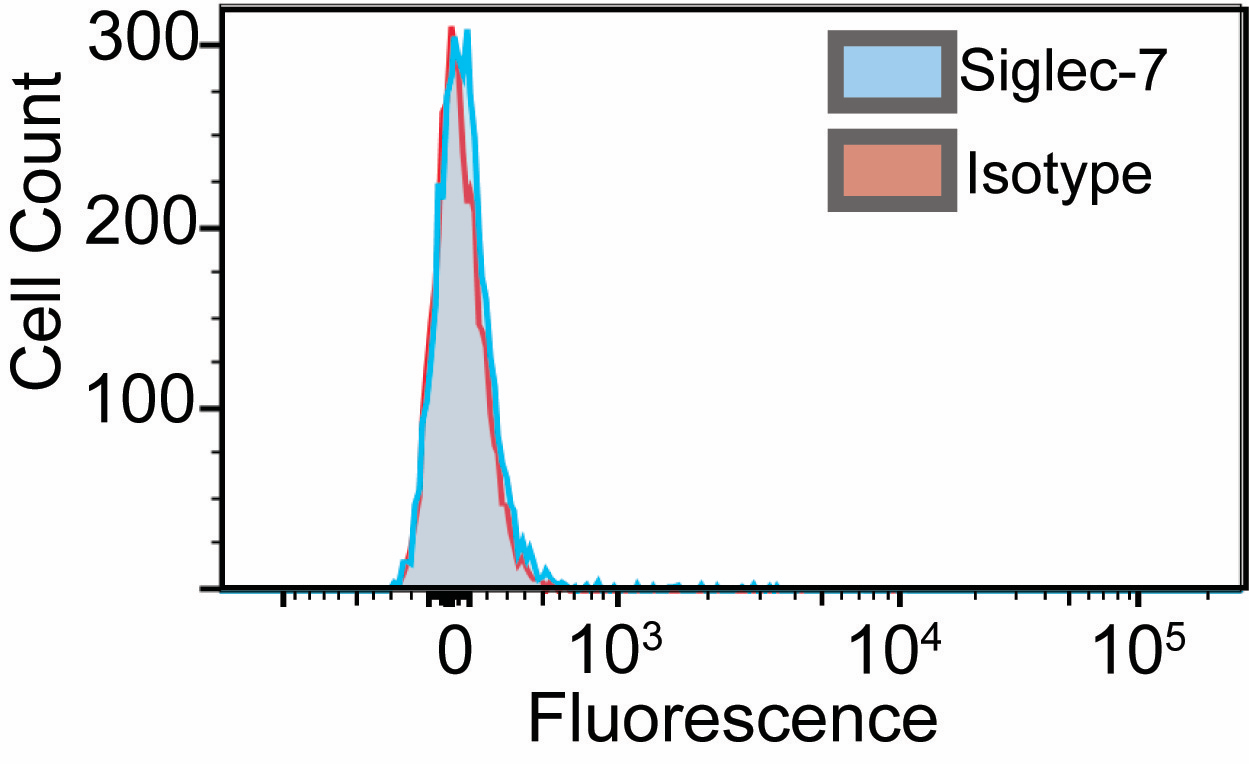
**

**Supplementary Figure 8.** Representative flow cytometry plot showing expression of the Siglec-7 receptor (S7.7 antibody clone, BioLegend) on peripheral CD3+ T-cells. Image is representative of 4 different donors.


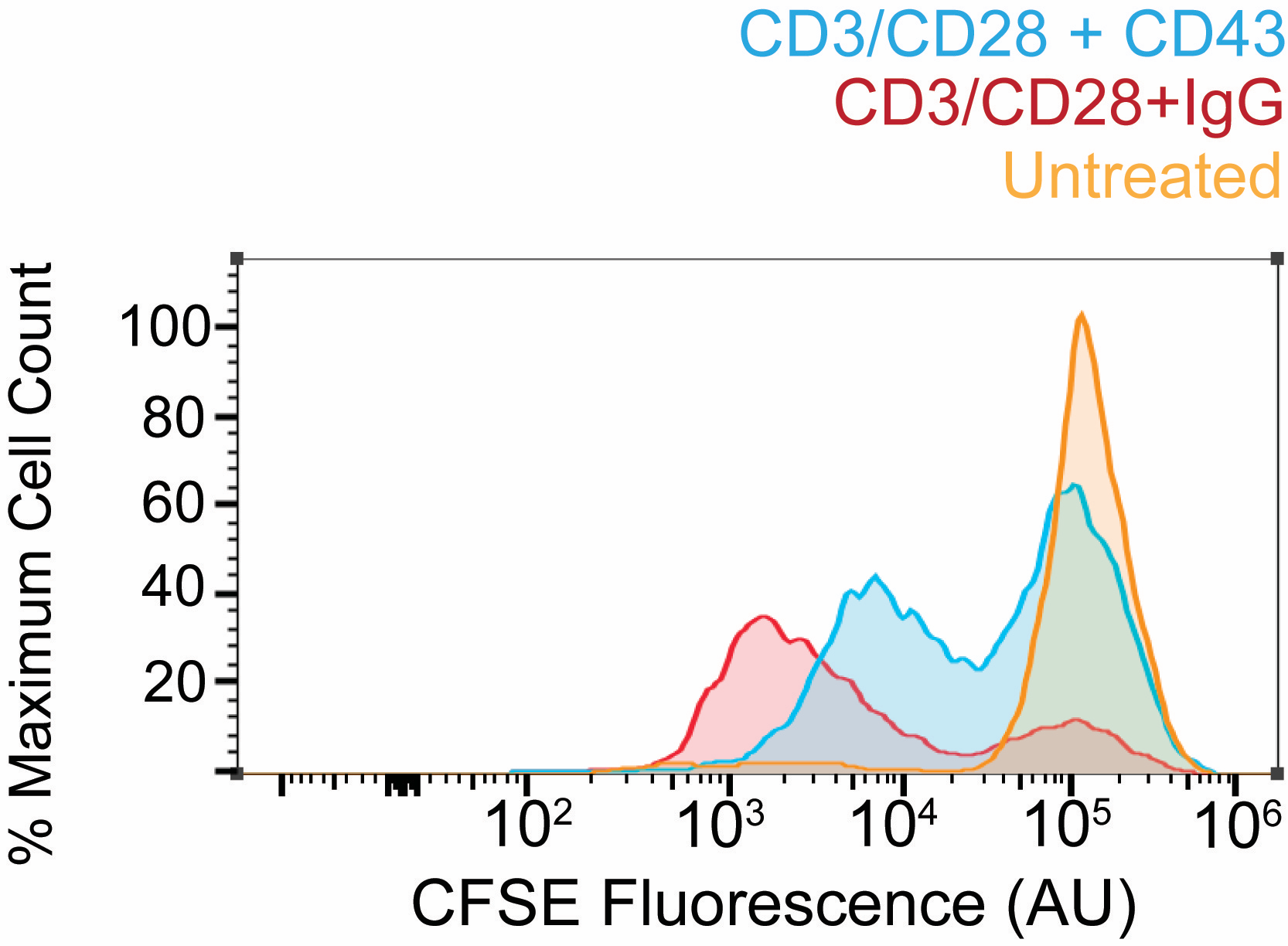


**Supplementary Figure 9.** Representative flow cytometry plot showing T-cell proliferation (as measured by CFSE staining) following treatment with both CD3/CD28 and anti-CD43 (10 μg/mL) antibodies.
